# Hypocapnia Stimuli-Responsive Engineered Exosomes Delivering miR-218 Facilitate Sciatic Nerve Regeneration

**DOI:** 10.3389/fbioe.2022.825146

**Published:** 2022-02-08

**Authors:** Yingshuai Wang, Tao Yu, Feihu Hu

**Affiliations:** School of Lifescience and Technology, Weifang Medical University, Weifang, China

**Keywords:** peripheral nerve regeneration, hypocapnia environment, engineered exosomes, delivering miR-218, nerve tissue engineering

## Abstract

Therapeutic strategies of microRNAs (miRNAs) and exosomes have been systematically explored as an enhancing application by paracrine and modulating cellular activity after internalization of recipient cells *in vitro*, and progressively developed to meet the requirements of peripheral nerve regeneration *in vivo*. However, how to obtain exosomes with superior properties and effectively deliver miRNAs becomes a key challenge. Hypocapnia environment might play unexpected outcomes in strengthening exosome function when culturing adipose-derived stem cells (ASCs). Previously, we discovered the intensive regulation of miR-218 on the differentiation of ASCs. In the present study, we analyzed the functional differences of secreted exosomes in response to hypocapnia stimulation, and explored the application in combination with miR-218 to facilitate sciatic nerve regeneration. Our results indicated that the delivery system of engineered exosomes derived from ASCs remarkably loads upregulated miR-218 and promotes cellular activity in the recipient cells (PC12 cells), and hypocapnia stimuli-responsive exosomes exhibit strengthening properties. Furthermore, in a sciatic nerve injury model, exosomes delivering miR-218 combined with engineered scaffold facilitated the regeneration of injured sciatic nerves. In the hypocapnia-stimulated exosome group, more encouraging promotion was revealed on the regeneration of motor and nerve fibers. Hypoc-miR-218-ASC exosomes are suggested as a promising cell-free strategy for peripheral nerve repair.

## Introduction

Peripheral nerves have a limited capacity to self-repair, and repair of severe damage or serious defects is often clinically unsatisfactory, with less than 50% of patients regaining full functional recovery after surgical treatment (Kehoe et al., 2012). Although end-to-end anastomosis or bridge-gap grafts can be the gold standard for curative reconstruction, many novel therapeutic approaches including microRNA (miRNA) strategies are currently being developed and explored in basic, preclinical, and clinical trials. MiRNAs are acknowledged to be key regulators of various cellular processes by interfering with protein expression and mRNA degradation. Existing studies confirm the notable differences in miRNA profiles between autologous repair and tissue engineering-assisted regeneration. The intrinsic mechanisms and molecular targets have been extensively delved (Yousefi et al., 2019). For example, in the initial period of nerve injury, the expressions of miR-204, miR-27a, and miR-29b were continuously down-regulated, while those of miR-182, miR-221/222, and miR-27a were reversed. MiR-30a, miR-124, miR-9, and miR-132 were wavily expressed during scaffold, facilitating the Schwann cells’ migration, axonal regeneration, and myelination (Yu et al., 2015; Tang and Sun, 2020). In our previous studies, miR-218 could promote the differentiation of mesenchymal stem cells (MSCs) to neuronal cells by spatiotemporal induction, and cell-engineered grafts facilitated peripheral nerve regeneration in combination with tissue engineering scaffolds (Hu et al., 2017b; Hu et al., 2017c).

Additionally, the delivery and release of exogenous miRNAs can maximize the application and effectiveness of this therapeutic approach, and serve as an alternative strategy for peripheral nerve regeneration. As a novel and exciting delivery vehicle, exosomes therein have garnered widespread attention. Exosomes are extracellular vehicles and natural nanocarriers derived from various cell types, and can load bimolecular cargoes such as bioactive proteins, mRNAs, and miRNAs to mediate cell-to-cell communication. The intrinsic bioactivity helps to traverse the biological barrier and improve the steadiness in bloodstream. In comparison with artificial nanocarriers, the longer circulation time prolongs their absorption potential and medicinal effectiveness on targeted tissues and cells *in vitro* and *in vivo*. The wide sources, stable storage, easy modification, negligible rejection, and lower risk of tumorigenesis support the exosome delivery system as a new mode of cell-free therapy (Tian et al., 2014; Zhou et al., 2017; Huang et al., 2021; Park and Seo, 2021; Shafiei et al., 2021).

Inspired by this, specific miRNAs can be encapsulated into exosomes and transported to the site of lesion and injury. Exosomes delivering miR-126 promoted angiogenesis and reduced apoptosis after spinal cord injury (Huang et al., 2020). The microglial exosomal miR-124-3p inhibited neuronal inflammation in scratch-injured neurons (Yang et al., 2019). As a cargo, miR-17-92 cluster down-regulated protein kinase B and glycogen synthase kinase 3β, and accelerated myelin remodeling after delivery by exosomes of MSCs (Xin et al., 2017a).

It is noteworthy that the characteristics of exosomes vary with the conditions of their production. Lower gas environment enriched Zeb2/Axin2 to exosomes, and further alleviated axonal demise and promoted synaptic proliferation *in vitro* (Wei et al., 2021). Under the same conditions, the release of exosomes loaded with miR-133b was significantly increased, and the intra-arterially injection additionally enhanced neurons’ elongation and brain plasticity in a cultured cortical embryonic rat (Xin et al., 2017b).

Due to this apparent discrepancy, in this study, we focused on the characteristic differences of exosomes derived from ASCs while culturing secretory cells in a low CO_2_ environment (3% CO_2_ conditions) and in the general environment (5% CO_2_ conditions), and assessed the delivery ability of miR-218. We aimed to reveal the properties of stimuli-responsive exosomes upon transfer from ASCs to PC12 cells. The exploration of phagocytosis, migration, and proliferation helps to characterize the facilitation of cellular activity by exosomes.

In combination with injected exosomes, we fabricated a cell-free poly(3-hydroxybutyrate-co-3-hydroxyvalerate) (PHBV) nano-fiber scaffold and transplanted it into a sciatic nerve injury model. The efficacy on injured neural tissue was exposed by motor functional restoration and histological assays. Our encouraging results help to explore the application of exosomes *in vivo*, especially hypocapnia environmental stimuli-responsive engineered exosomes. This study provides a promising cell-free approach for future clinical miRNA therapeutic strategies.

## Materials and Method

### Cell Culture

ASCs were extracted from adipose tissue as previously described (Zuk et al., 2002). The animal study was reviewed and approved by the Medical Ethics Committee of Weifang Medical University in China (no. 2019141). Briefly, the inguinal subcutaneous fat tissue was isolated from Sprague–Dawley (SD) rats. After digestion with 0.1% collagenase I and II (Sigma-Aldrich, United States) for 2 h, the cells were centrifuged and cultured with Dulbecco’s modified Eagle medium (DMEM; Thermo Fisher Scientific, United States) with 5% fetal bovine serum (FBS; Thermo Fisher Scientific, United States) at 37°C in a 5% CO_2_ environment. The medium was changed every 3 days, and cells were passaged for subsequent studies until 80% confluence was reached.

### Isolation and Characterization of Exosomes

**FIGURE 1:**
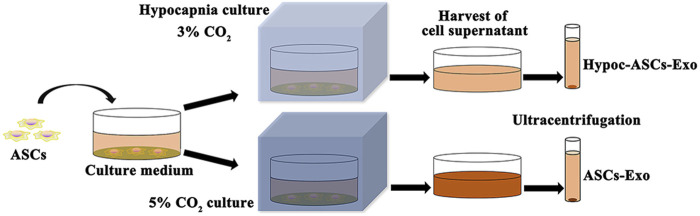
Schematic representations of exosomes isolated from two cultured environments.

Herein, to remove the interference of FBS, ASCs were cultured in a specially treated basal medium (added FBS was vesicle-depleted by an overnight ultracentrifugation at 110,000 g), and exosomes were purified by ultracentrifugation (Liang et al., 2011). The hypocapnia stimuli-responsive exosomes were achieved as follows: ASCs were cultured in a hypocapnia environment (3% CO_2_ conditions) for 48 h, and the first culture medium was harvested. The fresh basal medium was subsequently added and maintained for 48 h to form a second culture medium. All mediums were collected for the isolation of exosomes. Meanwhile, the medium of ASCs cultured in the general environment (5% CO_2_ condition) was served as control. The mediums were centrifuged at 300 × g for 5 min to eliminate cells, and 10,000 × g for 30 min for removing debris of cells. Finally, the supernatant was centrifuged at 100,000 × g (ultracentrifugation) for 8 h twice for purification of exosomes by L-100 XP ultracentrifuge (Beckman Coulter, United States) (Figure 1). The purified exosomes (Hypoc-ASCs-Exo group and ASCs-Exo group) were resuspended into PBS and stored at −80°C.

The morphology of exosomes was observed by transmission electron microscopy (TEM). Exosomes were transferred onto a carbon-coated grid, kept at room temperature for 20 min, and then visualized under TEM (JEM-2100, JEOL, Japan). The vesicle size distribution was characterized by dynamic light scattering (DLS, Zetasizer Nano ZS, United Kingdom). The specific markers were confirmed as Supplementary Figure S1.

### Construction of miR-218 Plasmids and Exosomes Delivering miR-218

MiR-218 plasmids were designed as shown in Figure 3. The multiple cloning site of pPG-eGFP-miR plasmid was inserted with miR-218 objective genes (sense strand), loop, and antisense strand. Enhanced green fluorescence protein (eGFP) was used for a reporter. ASCs were seeded onto 35 mm dish and incubated until 80% confluence, and 10 µl Superfectin Transfection Reagent (Qiagen, Canada) and miR-218 or miR-NC plasmids were added to the medium for transfection. After 24 and 48 h, positive expressions of eGFP were assayed by microscopic fluorescence to identify transfection efficiency (Ti-U Nikon, Japan).

ASCs of highly expressed miR-218 were cultured in hypocapnia and general environments, and the specific exosomes delivering miRNAs were isolated as described above. Expression levels of miR-218 were measured by quantitative real-time PCR in untreated ASCs (Control group), miR-218-transfected ASCs (miR-218-ASCs group), isolated exosomes from generally cultured miR-218-transfected ASCs (miR-218-ASCs-Exo group), and isolated exosomes from hypocapnia-cultured miR-218-transfected ASCs (Hypoc-miR-218-ASCs-Exo group).

### PC12 Cells Activity After Internalizing the Exosomes Delivering miR-218

Cellular uptake and distribution of exosomes in PC12 cells were visualized by labeling of fluorescent dye 3,3′-dioctadecyloxacarbocyanine perchlorate (DiO, beyotime, China) under a microscope. Samples (miR-218-ASCs-Exo and Hypoc-miR-218-ASCs-Exo) were incubated with 5 µM DiO at 37°C for 20 min. After two cycles of washing-centrifugation, exosomes were resuspended in PBS. As a widely neural model, PC12 cells were used to expose the deliverable function of exosomes (Westerink and Ewing, 2008). The distribution of DiO-labeled exosomes was analyzed as follows: PC12 cells were seeded onto 35 mm dish and incubated until 80% confluence. Cells were then incubated with the basal culture medium supplemented with 5 µg/ml DiO-labeled exosomes for 24 h. Next, cell nuclei were stained with Hoechst 33342 (Shanghai Shenggong Co., Ltd., China), and fluorescence images were recorded by the confocal fluorescence microscope (Zeiss LSM710, Germany).

The scratch assay was applied to expose the migratory characteristics of PC12 cells’ response to two exosomes (miR-218-ASCs-Exo and Hypoc-miR-218-ASCs-Exo groups) as follows: PC12 cells were seeded onto 35 mm dish and incubated until 80% confluence, and a wound maker scratched across to form a physical gap. The two groups of exosomes of 5 µg/ml were added to the scratched cell medium. After 24 h, the scratched images of PC12 cells were microscopically recorded. The transwell assay further assessed the cytotaxis of PC12 cells. Briefly, the PC12 cells placed on the upper layer could be driven across the cell-permeable membrane (8 μm aperture) by the exosomes in the lower layer. After 48 h, the cells were stained by Hoechst 33342 and counted in a view field. Furthermore, the mediums with different contents and sources exosomes (the miR-218-ASCs-Exo and Hypoc-miR-218-ASCs-Exo of 5 µg/ml, 10 µg/ml, and 20 µg/ml) were used to culture PC12 cells (the untreated cells were served as a control), and cell viability was evaluated by the Cell Counting Kit-8 method (Dojindo, Kumamoto, Japan) at 24 and 48 h.

### Quantitative Real-Time PCR

The expression levels of miR-218 and targeted genes (*Robo 1*, *Robo 2*, *Sfrp 2*, and *Dkk 2*) were examined by qRT-PCR. The RNA was isolated from un-treated PC12 cells (Control group), PC12 cells transfected miR-218 (miR-218 group), and PC12 cells treated by two kinds of exosomes (miR-218-ASCs-Exo and Hypoc-miR-218-ASCs-Exo groups). According to the M-MLV reverse transcriptase instructions, RNA was converted to cDNA. The PCR reactions were performed using One-step 2 × SG Green qRT-PCR Mix (TaKaRa, China) on the ABI 7500 system (Application Biosystems, United States). The *U6* and *Gapdh* were used as housekeeping genes.

### Preparation of Nanofibrous Scaffold and Animal Surgical Procedures

Herein, the PHBV nano-fiber films were obtained *via* the electrospinning technique as previously described (Hu et al., 2017a). In brief, PHBV (no. 403121, Sigma-Aldrich, Germany) was dissolved in TFE (2,2,2-trifluoroethanol, Guangzhou Darui Company, China) to form a 2% (w/v) solution. The nano-fiber films were fabricated by the TL-01 type electrospinning apparatus (Shenzhen TongliWeina Technology Company, China). The biocompatibility were detected as Supplementary Figures S2, S3.

PHBV nanofibrous scaffolds were gifted to a rat sciatic nerve injury model *in vivo*. First, the films were rolled into a hollow tubular structure. In surgical procedures, SD rats were anesthetized, and sciatic nerves were exposed and truncated. The ends of segment were inserted into the lumen of conduit, and the incision was closed with silk sutures. Animals were fed *ad libitum* and kept in a standard environment. After 2 days, rats (*n* = 15) were randomly divided into three groups. Intravenous injections of 2 mg exosomes were performed for each rat *via* tail vein every 3 days continuously for 4 weeks (PHBV-miR-218-ASCs-Exo group and PHBV-Hypoc-miR-218-ASCs-Exo group). Rats of PHBV group were injected with PBS.

### Sciatic Function Index and Gastrocnemius Muscle Analysis

The recovery of motor function was analyzed by the sciatic function index (SFI) (Bain et al., 1989). The lengths of the third toe to heel (PL), the first to fifth toe (TS), and the second to fourth toe (IT) were measured on the experimental side (E) and contralateral normal side (N) in each rat. The SFI value was calculated by the following formula: SFI = 109.5* (E’TS−N’TS)/N’TS+13.3*(E’IT−N’IT)/N’IT−38.8*(E’PL−N’PL)/ N’PL−8.8. The 0 value represents normal nerve function, and around −100 value represents total dysfunction. At weeks 4 and 8, three groups of rats were allowed to walk across a 100-cm long channel in order to record footprints along the way. At the end of research, the gastrocnemius muscle of three groups was dissected and weighed on an electronic balance (while still wet). The atrophy of gastrocnemius muscle was calculated by wet weight of injured limb and control (100% ratio).

### Histological Examination

Gastrocnemius muscle and regenerated nerve were stained and analyzed to evaluate the recovery of muscle, nerve fiber, and myelin sheath. Muscles and central segment nerves were fixed in 10% buffered formalin for 48 h. Samples were dehydrated through a graded ethanol series, cleared in xylene, and cut into 5-μm-thick sections after paraffin embedding. Transverse sections of gastrocnemius muscle were stained with Masson trichrome, and nerve samples were stained with hematoxylin and eosin (H&E) and observed by microscope.

### Statistical Analysis

The results were expressed as the mean ± SD. Statistical analysis was performed using GraphPad Prism software.

## Results

### Characterization of ASCs-Exo

**FIGURE 2:**
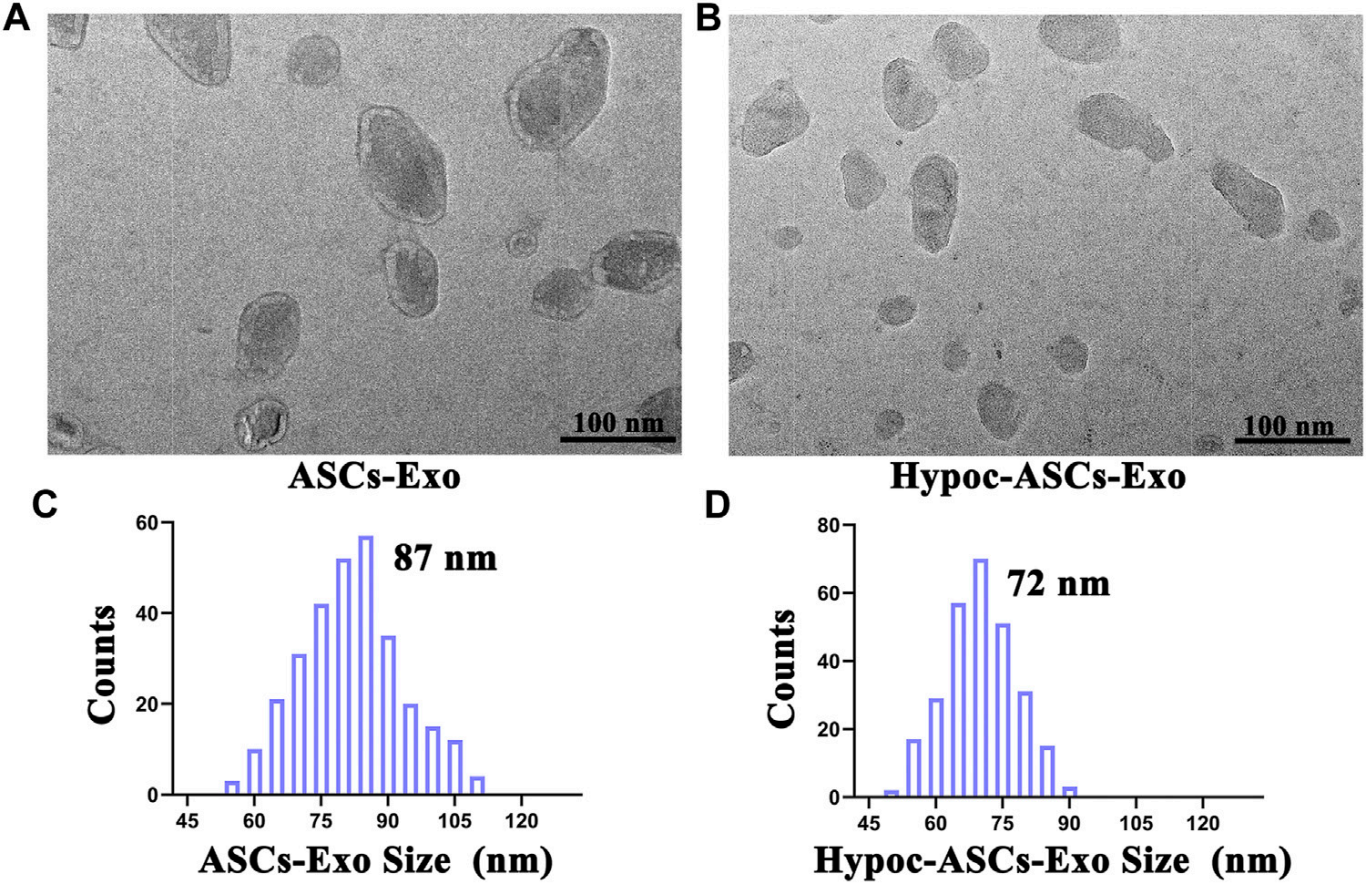
Morphology and diameter distribution of ASCs-Exo and Hypoc-ASCs-Exo. **(A,B)** Morphology of exosomes were assessed by TEM (scale bars = 100 nm). **(C,D)** Diameter of the exosomes was assessed by dynamic laser scattering (DLS).

Exosomes were isolated from cell supernatant by ultracentrifugation and characterized using TEM and DLS (Figures 2A–D). Photographs revealed that the ASCs-Exo group has a typical saucer-like bilayer membrane structure with an average diameter of 87 nm. Notably, TEM images indicated a few moderate morphological differences compared to hypocapnia environment-derived products, such as slightly smaller average diameter (72 nm) in the Hypoc-ASCs-Exo group. DLS assays were consistent with TEM morphology in terms of size distribution. The morphologies and size distribution supported that the hypocapnia environment can also stimulate ASCs to secrete exosomes with noteworthy differences of physical size from those obtained in the general environment.

### Construction and Validation of miR-218-ASCs-Exo

**FIGURE 3:**
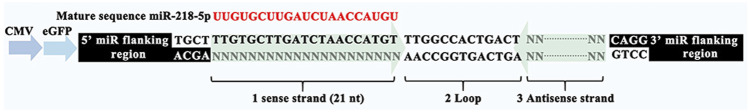
Design and construction of miR-218-5p plasmid.

**FIGURE 4:**
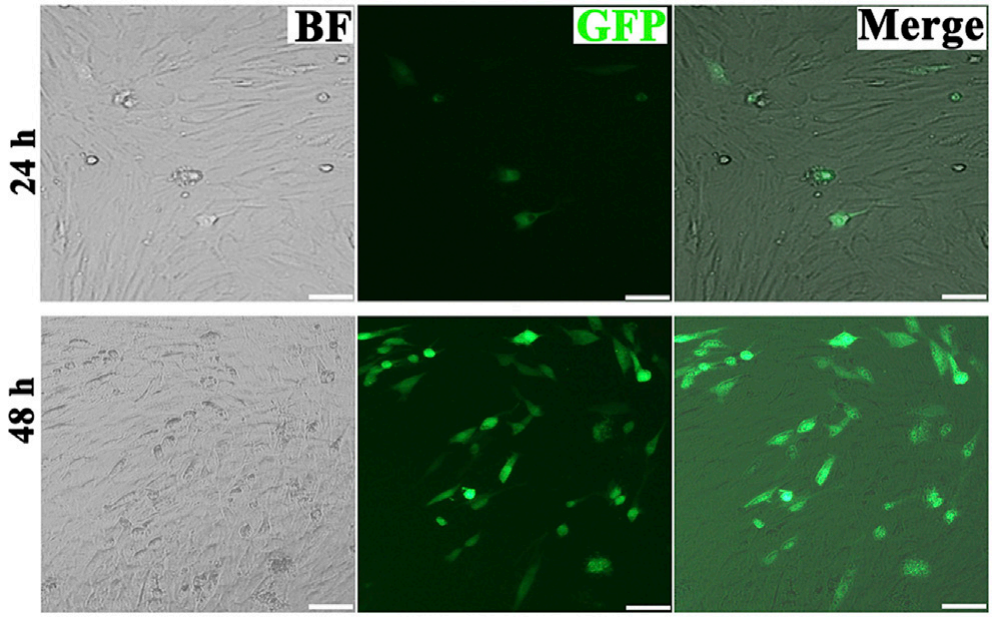
Micrographs of fluorescent, bright field, and merged images of transfected ASCs after 24 and 48 h (scale bars = 20 μm).

**FIGURE 5:**
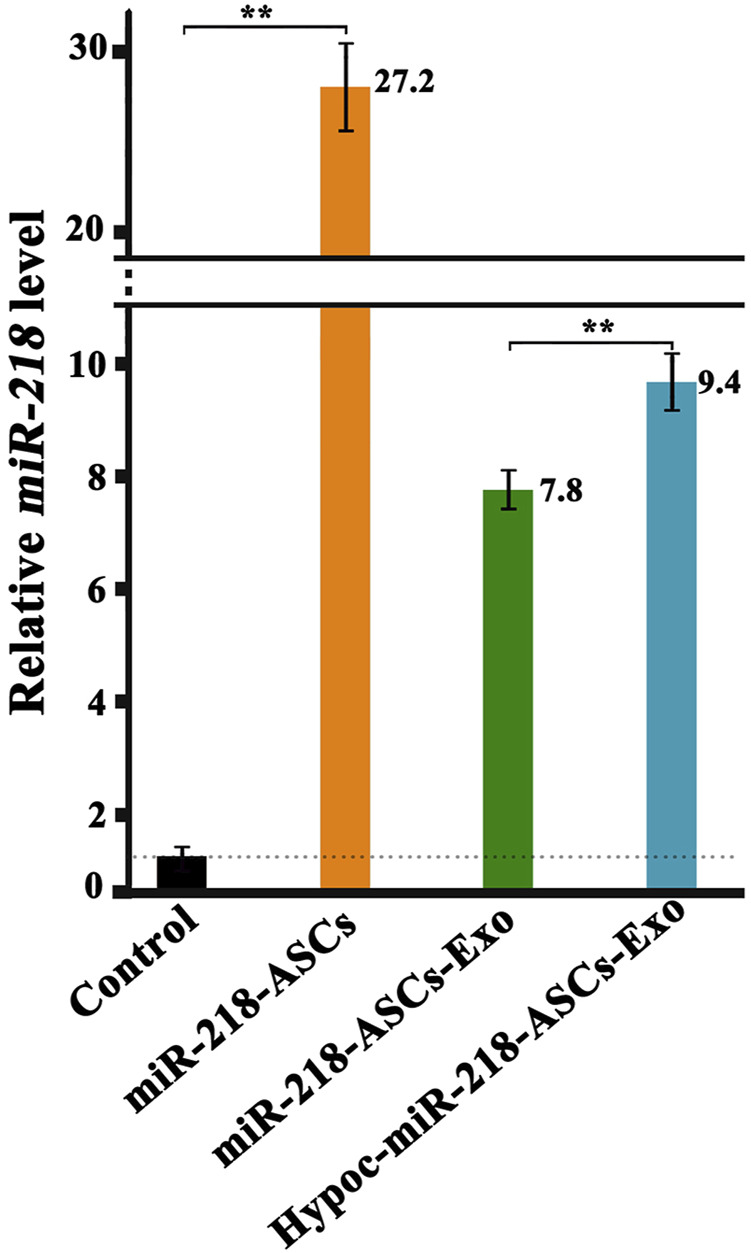
Expression levels of miR-218 in untreated ASCs (normalization and served as the Control group, C group), transfected ASCs (miR-218-ASCs group), exosomes isolated from general environment cultured the transfected ASCs, and exosomes isolated from hypocapnia environment cultured the transfected ASCs.

**FIGURE 6:**
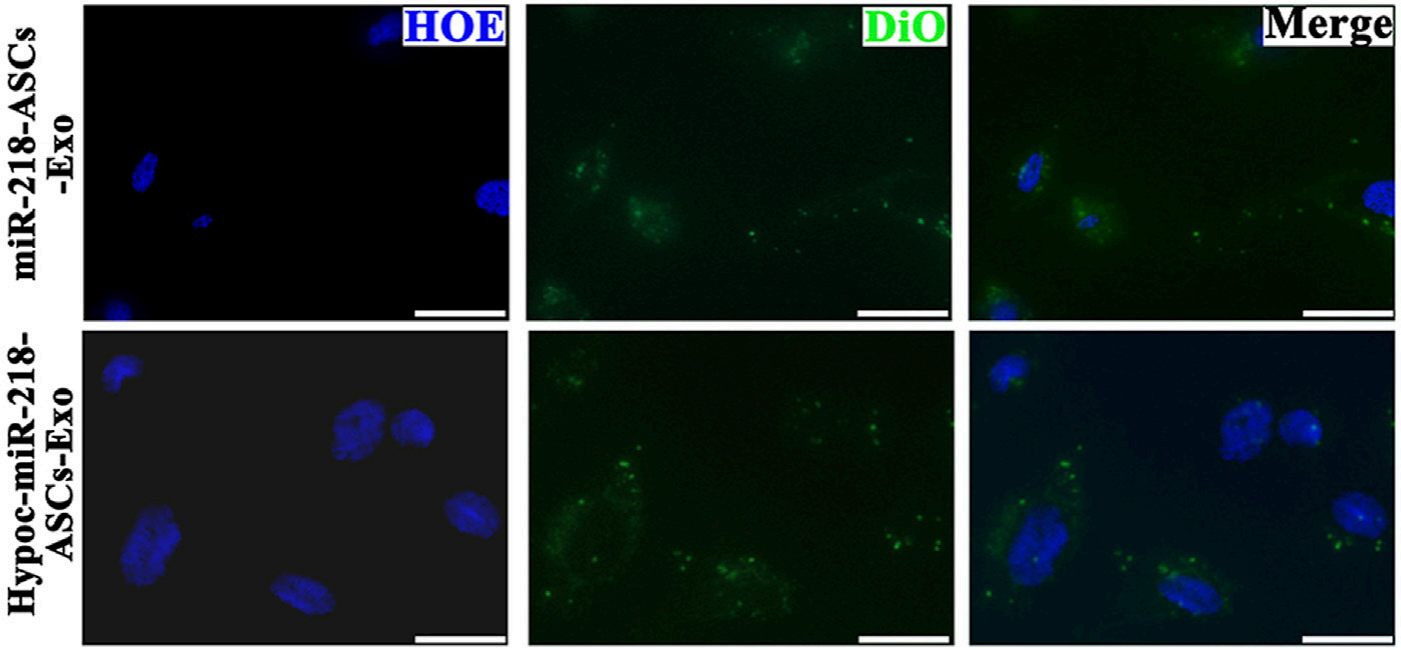
Confocal images of DiO-labeled exosomes (green) and nuclei (blue) were monitored by microscope in two groups (scale bars = 10 μm).

To verify whether exosomes can concentrate and deliver miR-218, and evaluate the miR-218-regulative strategy, we constructed an expressed plasmid of miR-218 *in vitro* (Figure 3). The appearance and increase of eGFP-positive cells proved the successful expression of miR-218 plasmid in ASCs (Figure 4). qRT-PCR measured the expression levels of miR-218 in four groups. Compared to the baseline of the miR-218 expressed level in untreated ASCs (Control group), ASCs transfected with miRNA plasmid had 27.2-fold upregulation. As cytoplasmic secretion products, exosomes of two groups highly packed the mature miR-218 (7.8-fold in miR-218-ASCs-Exo group and 9.4-fold in Hypoc-miR-218-ASCs-Exo group) (Figure 5). Next, the phagocytosis of PC12 cells was used to analyze the delivery properties of exosomes between different cells. The clear and perinuclear dotted staining confirmed that exosomes isolated from ASCs are successfully transported to the cytoplasm of PC12 cells as expected, highlighting similarities regardless of miR-218-ASCs-Exo or Hypoc-miR-218-ASCs-Exo groups (Figure 6).

### Enhancement of PC12 Cells Viability by Internalized miR-218-ASCs-Exo and Hypoc-miR-218-ASCs-Exo

**FIGURE 7:**
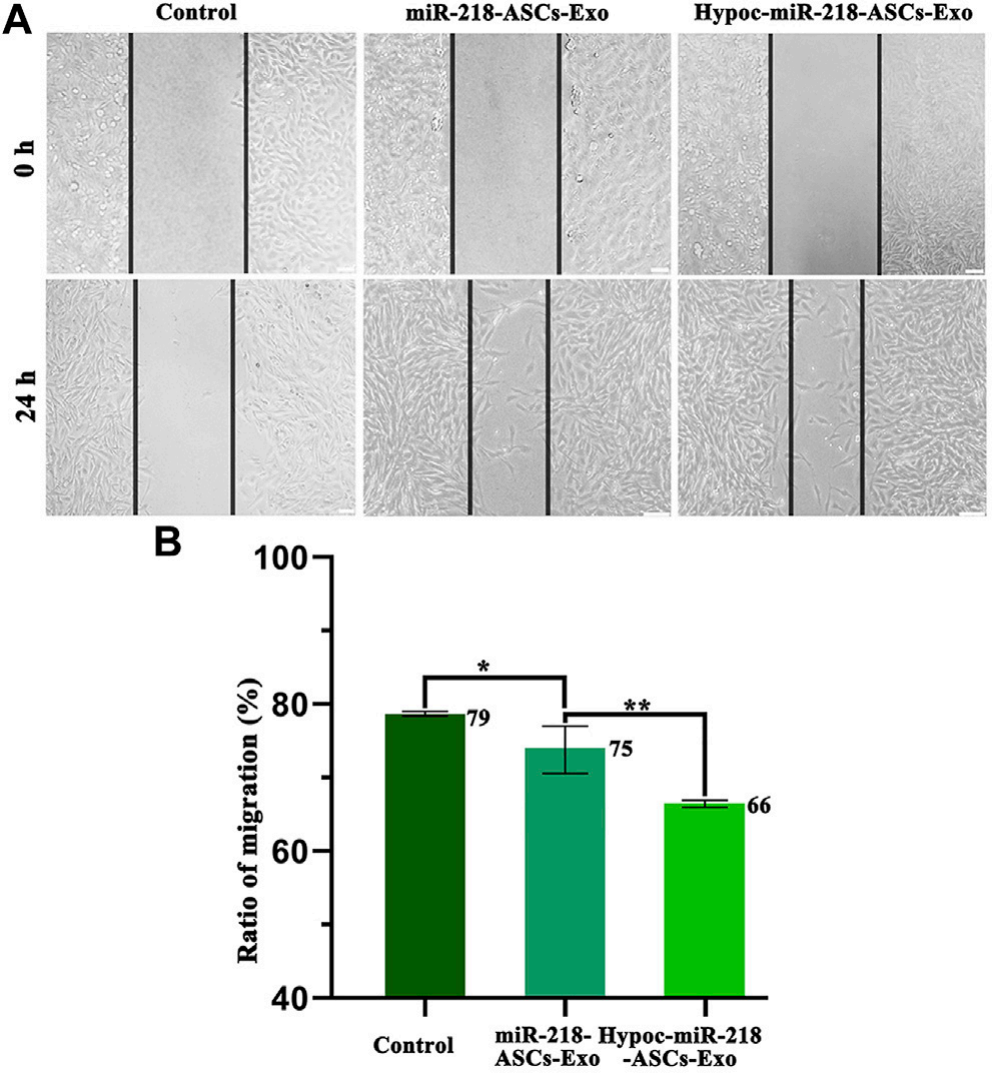
Scratch assay and ratio of migration. **(A)** Images of control (untreated PC12 cells’ group), miR-218-ASCs-Exo (miR-218-ASCs-Exo-treated PC12 cells’ group), and Hypoc-miR-218-ASCs-Exo (Hypoc-miR-218-ASCs-Exo-treated PC12 cells’ group) at 0 and 24 h. **(B)** Ratio of migration (%) was calculated in three groups, respectively, at 24 h.

**FIGURE 8:**
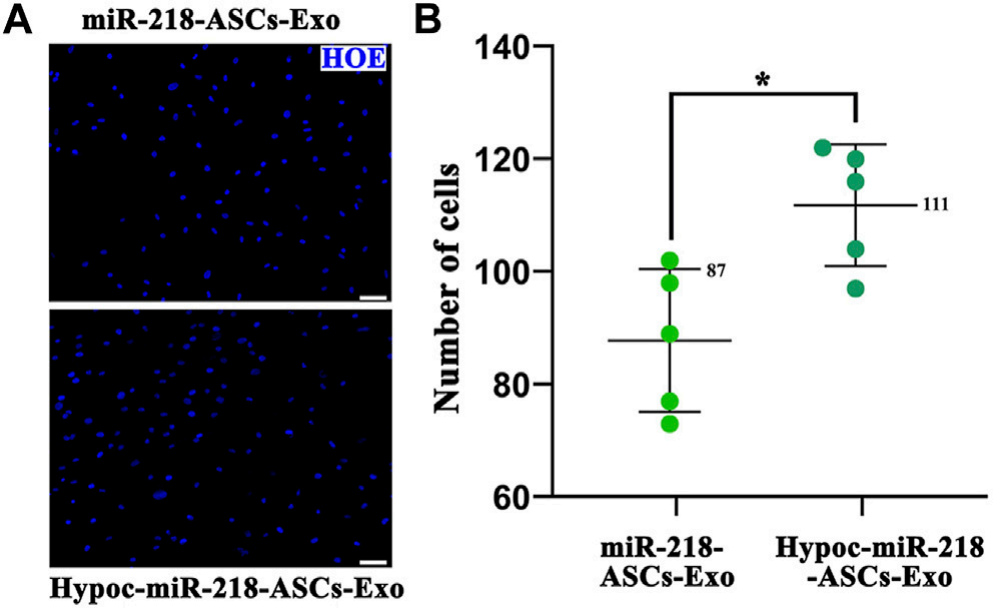
Transwell assay. **(A)** Nuclei of the cell across the membrane were stained by Hoechst 33342 (blue) (scale bars = 50 μm). **(B)** Cell number in a view field was counted, respectively, in two groups.

**FIGURE 9:**
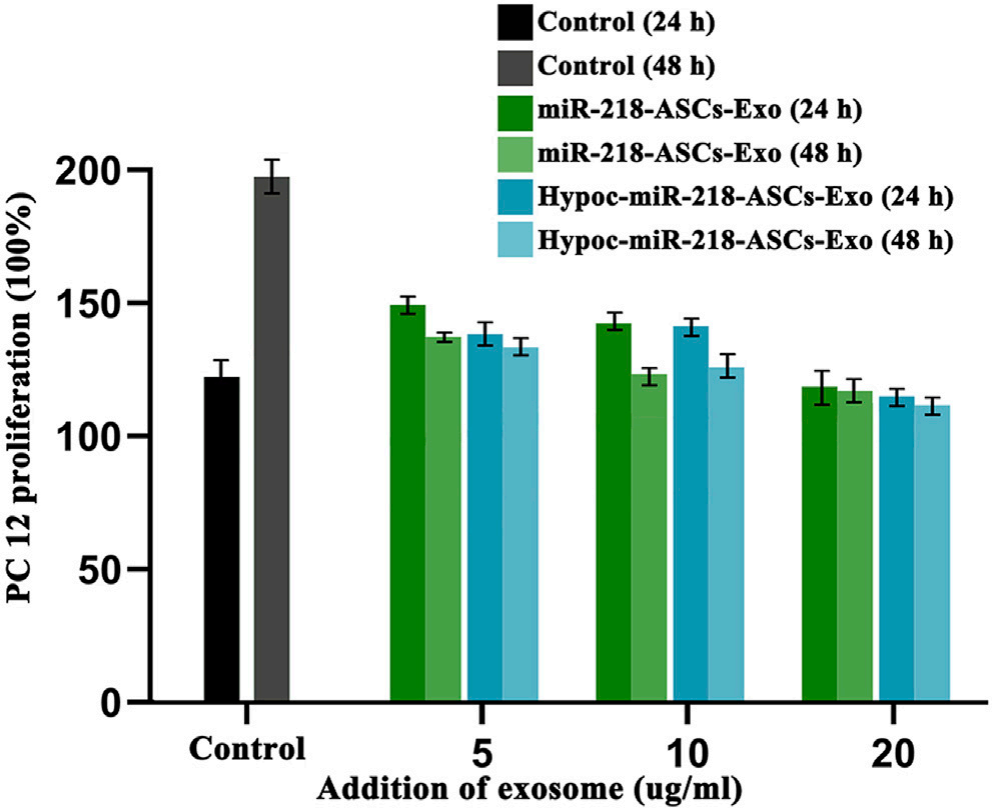
PC12 proliferation was tested after the cultured medium was added to 5 µg/ml, 10 µg/ml, and 20 µg/ml exosomes. Untreated PC12 cells served as the Control group. Expression levels of miR-218 targeted genes (*Robo 1*, *Robo 2*, *Sfrp 2*, and *Dkk 2*) were examined by qRT-PCR in untreated PC12 cells (Control group), PC12 cells of transfected miR-218 plasmid, and two exosomes groups.

The wound-healing approach exposed the migration characteristics of PC12 cells after internalizing exosomes. After 24 h, compared to group of non-exosomes added to the medium (control group), quantitative analysis of scratched distance exhibited a statistically significant shrinkage in the Hypoc-miR-218-ASCs-Exo and miR-218-ASCs-Exo treatment groups (Figures 7A,B). In the transwell tests, the Hypoc-miR-218-ASCs-Exo group showed a higher number of nuclei stained with 111 cells than 87 cells in a view field (Figures 8A,B). The above results confirmed that Hypoc-miR-218-ASCs-Exo increases PC12 viability and promotes migration after the internalization of exosomes. Furthermore, cell proliferation results observed that the addition of exosomes may inhibit cell division and proliferation, and with the increase of dose and duration, the differences between groups are continuously widened in a dose-dependent manner. The Hypoc-miR-218-ASCs-Exo-treated PC12 cells performed differential property *versus* the miR-218-ASCs-Exo group (Figure 9).

### Targeting of miR-218 Delivered by Exosomes

**FIGURE 10:**
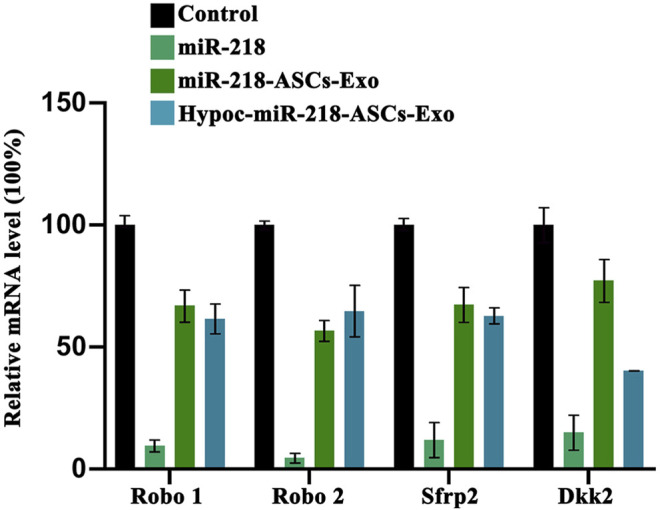
Expression levels of miR-218 targeted genes (*Robo 1*, *Robo 2*, *Sfrp 2*, and *Dkk 2*) were examined by qRT-PCR in untreated PC12 cells (Control group), PC12 cells of transfected miR-218 plasmid, and two exosomes groups.

The levels of miR-218 target genes (*robo1* and *robo2*) and Wnt signaling antagonist genes (*sfrp 2* and *dkk2*) were validated by qRT-PCR. In comparison with untreated PC12 cells (Control group), exosomes of miR-218-ASCs-Exo or Hypoc-miR-218-ASCs-Exo groups could deliver miRNA to PC12 cells and suppress the expression levels of target genes (Figure 10), although with profound discrepancies to the directly transfected group (miR-218 group).

### Facilitation of Exosomes With PHBV Scaffold on Sciatic Nerve Recovery

**FIGURE 11:**
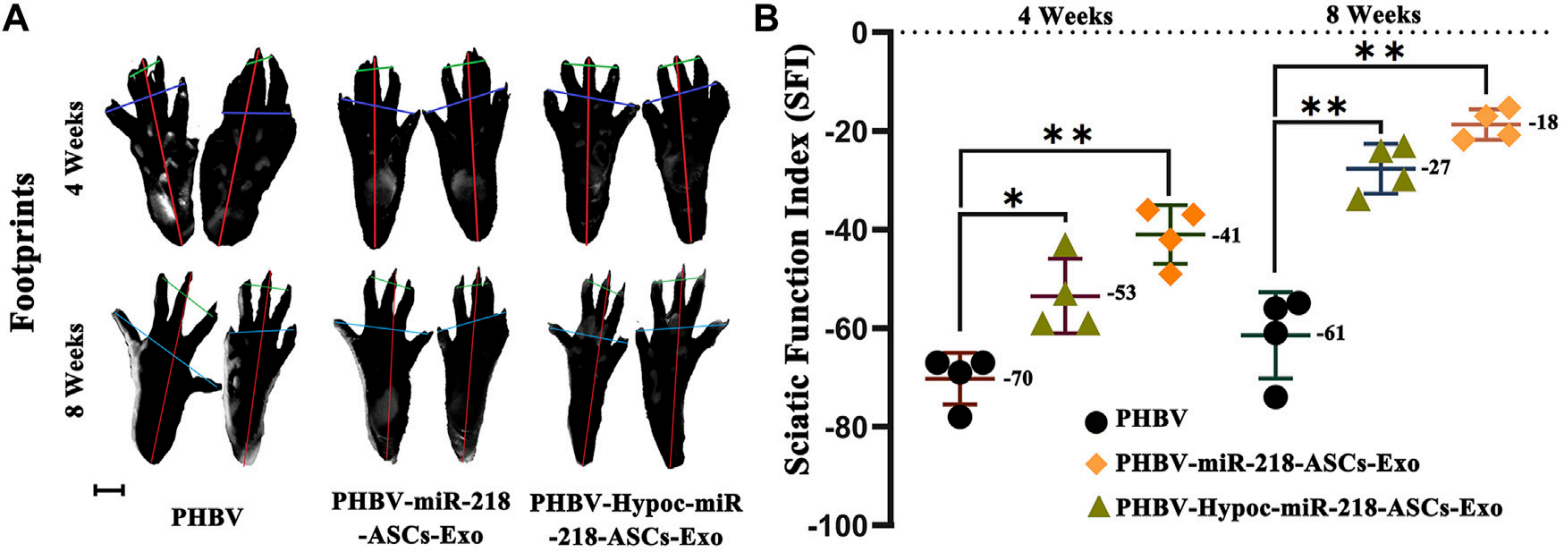
SFI of sole PHBV scaffold (PHBV group), scaffold combined with miR-218-ASCs exosomes (PHBV-miR-218-ASCs-Exo group), and scaffold combined with Hypoc-miR-218-ASCs exosomes (PHBV-Hypoc-miR-218-ASCs-Exo group) were tested after animal surgical procedures. **(A)** Footprints of three groups (scale bars = 2 mm). **(B)** SFI values of three groups.

**FIGURE 12:**
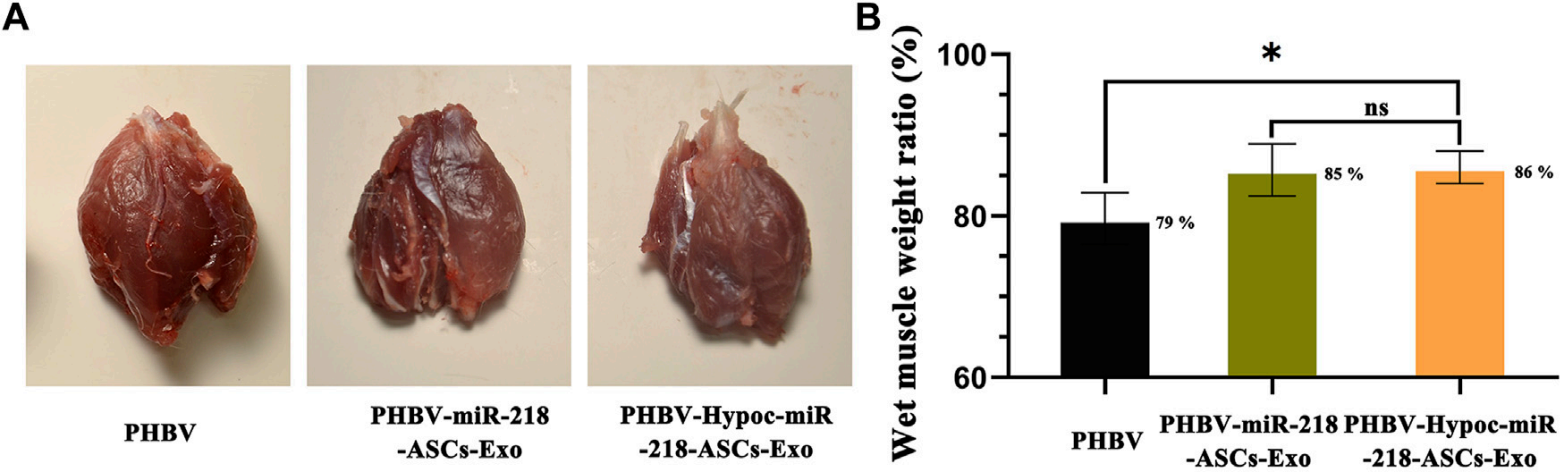
Wet weight of gastrocnemius muscles was tested after animal surgical procedures. **(A)** Images of gastrocnemius muscles of three groups. **(B)** Atrophy of gastrocnemius muscle was calculated by wet weight of injured limb and Control group in three groups.

We fabricated PHBV nanofibrous scaffold to facilitate the application of exosomes for the repair of sciatic nerve injury *in vivo*. After 4 and 8 weeks of surgery, the SFI values of PHBV-miR-218-ASCs-Exo and PHBV-Hypoc-miR-218-ASCs-Exo groups were significantly higher than the PHBV group (−41 and −53 compared to −70, and −18 and −27 compared to −61) (Figures 11A,B). The wet weight ratio of gastrocnemius muscle in the PHBV-Hypoc-miR-218-ASCs-Exo group was approximated to the PHBV-miR-218-ASCs-Exo group, showing difference with the Control group (PHBV group, 86 and 85% compared to 79%) (Figures 12A,B).

**FIGURE 13:**
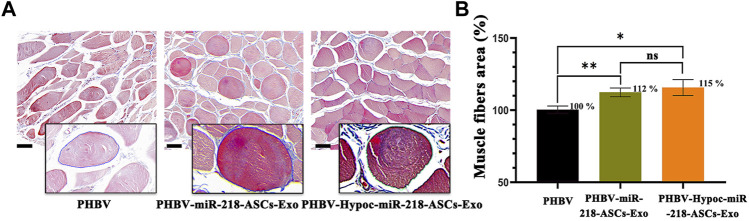
Masson trichrome staining of three gastrocnemius muscles groups. **(A)** Image of masson trichrome staining of three groups (scale bars = 40 μm). **(B)** Muscles fibers area was calculated.

**FIGURE 14:**
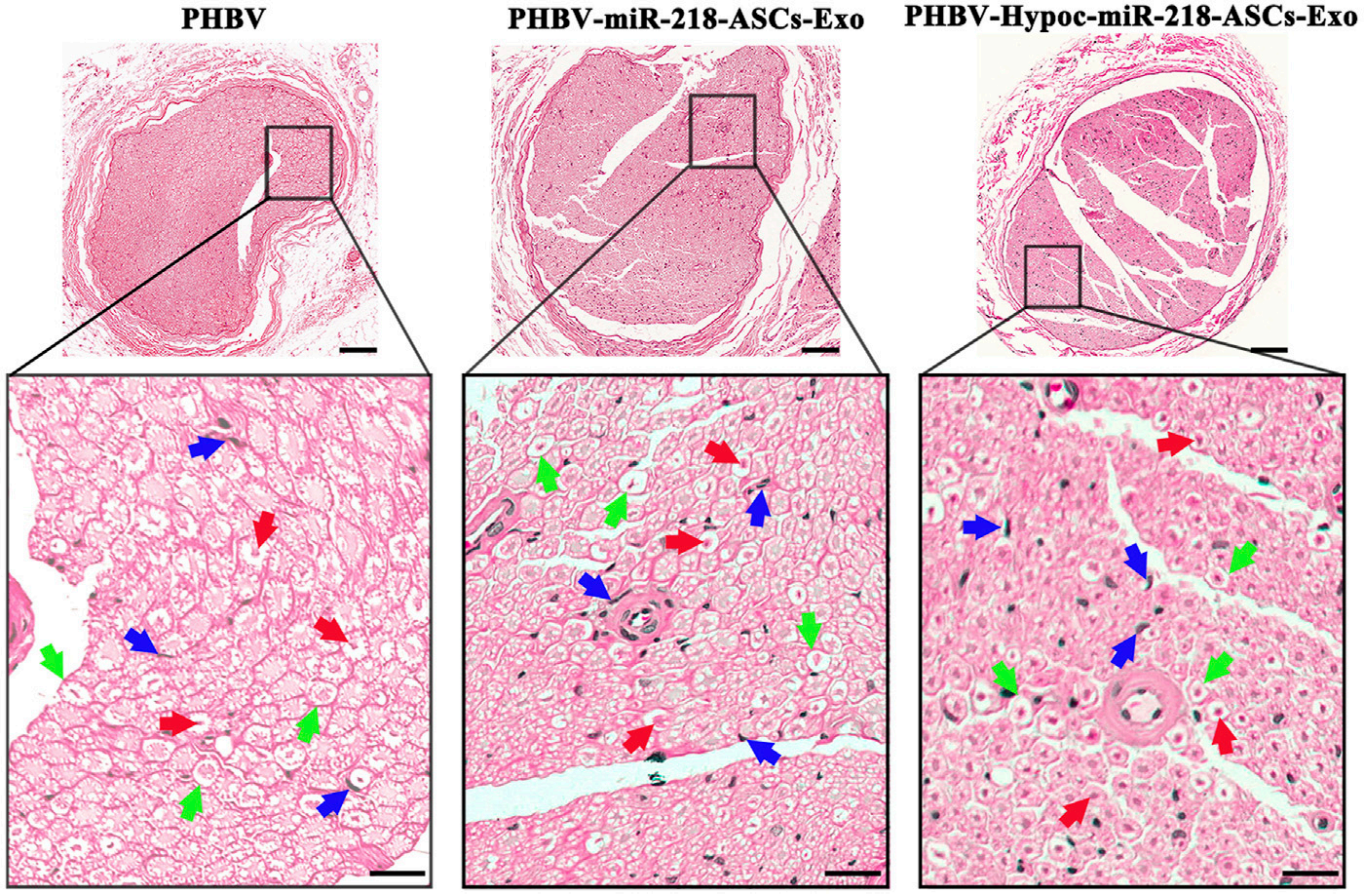
H&E staining of regenerated nerve tissue (scale bars = 100 μm), and red arrows pointed out axon, blue arrows pointed out SCs nucleus, and green arrows pointed out myelin sheath in regional enlarged drawing (scale bars = 20 μm).

The morphology of gastrocnemius muscles was similar to that of the normal muscles and showed slight atrophy of fibers in three groups (Figures 13A,B). However, the fibers area results supported the statistical difference in three groups (115 and 112% compared to 100%). In the regional enlarged drawing (Figure 14), histological examination of regenerated nerve fibers showed a dense structure with no significant difference in axon diameter or myelin thickness (green arrows). Conversely, more SC nuclei (blue arrows) were found in the PHBV-miR-218-ASCs-Exo group, especially in the PHBV-Hypoc-miR-218-ASCs-Exo group. Encouraging regenerated neural tissues supported that unite PHBV scaffold, the intravenous injections of exosomes, can promote nerve regeneration, and the exosomes stimulated and acquired by hypocapnia further adequately facilitate sciatic nerve recovery.

## Discussion

Tissue engineering therapies, including functional cell implants and cell-free physically engineered scaffolds, have been proven effective in promoting nerve regeneration. During repair, a variety of secreted factors are delivered to the injury sites and positively affect the survival and reproduction of neural cells, and a part of the agents is found to be enriched in exosomes. Significant discrepancies are observed in cellular exosomes from different sources, including the type and content of mRNAs, miRNAs, and proteins. This is an unavoidable obstacle in the clinical application of exosomes, and also provides opportunities for the preparation of personalized vectors. In the present study, we set out to investigate the application of miRNA therapy based on exosomes delivering miR-218 in sciatic nerve repair and systematically dissected the differential characteristics of exosomes when the secretory cells (ASCs) were cultured in a lower CO_2_ environment.

First, photographs indicated the successful isolation of exosomes. A slightly narrower size was noticed in Hypoc-ASCs-Exo. Regarding these alterations, we speculated that it was a consequence of hypocapnia environment, as all other procedures used to purify exosomes were identical. In our subsequent findings, it was further demonstrated that the smaller sizes in the Hyoc-ASCs-Exo group are more conducive to deliver miR-218, and assist miRNAs to perform a stronger regulatory function. Essentially, the physical properties of exosomes could influence the intercellular communication (Van Deun et al., 2014). Caponnetto et al. (2017) proved that the therapeutic use of exosomes can be enhanced by using size as a parameter to select functionally different sub-populations. Compared to the 81 nm, a smaller particle size distribution (43 nm) was more preferentially and rapidly absorbed by A172 human glioblastoma cells, triggering a stronger cellular motility response.

While exosomes loaded miRNAs, the selected cargoes were orchestrated. Green fluorescence confirmed the success of transfection even as primary culture cells, and ASCs undergo readily clonal distribution to resist transfection reagents. High levels of miR-218 in two groups distinctly indicated miR-218 are internalized and transferred from cytoplasm to the exocytosed exosomes. In tumors, cancer cells with high migration and activity are usually accompanied by high expression of miR-218, and upregulation inhibits the migratory ability (Zhu et al., 2016; Sun et al., 2021). In contrast, in the differentiation of MSCs, angiogenesis, hair regrowth, *etc*., upregulated miR-218 plays positive stimulators on cellular activity. These contradictory properties of miR-218 deserve attention. The almost filled gaps and higher numbers revealed that miR-218 seemingly promotes the activity of PC12. However, our proliferation assays performed the opposite indication. Combined with existing studies, a reasonable explanation was that upregulated or delivered miR-218 strengthens cellular activity and enhances the migration ability of PC12 cells rather than proliferation.

The exosomes delivering miR-218 suppressed the target gene (including the *Sfrp 2* and *Dkk 2*) in PC12 cells. The results were a hallmark of positive expression of Wnt signaling pathways. In the growth of hair shafts, the *β-Catenin* and T-cell-specific factor transcriptional activity were activated by miR-218 and targeted *Sfrp 2* (Hu et al., 2020). Melnik et al. exposed the dynamic governor of miR-218 and Wnt signaling on the hypertrophic degeneration of cartilage tissue after ectopic transplantation (Melnik et al., 2020). The abovementioned results suggested that exosomes delivering miR-218 (including Hypoc-miR-218-ASCs-Exo group) may have an enhanced role in the development and regeneration of nerve fibers *in vivo*.

At present, PHBV nano-fiber scaffold has been used as a support construction for cell growth in reconstructing damaged tissues or organs. In this study, the manufactured PHBV scaffold and injection of exosomes adequately helped sciatic nerve regeneration by a cell-free tissue engineering. Existing evidence discovered that during peripheral nerve repair, wound healing and nerve fiber re-extension and re-attachment are established on the physical elastic support and myelin wrap around the axons (Han et al., 2019). All processes might depend on the secretion of collagen, whereas they were exactly the target of miR-218. In gastric cancer, the expressions of collagen type I were correlated with miR-218 (Zhang et al., 2016). In Guo et al.’s research, overexpression of miR-218 suppressed the degradation of collagen type I and IV to overcome the homeostasis loss of connective tissue (Guo et al., 2019). Notably, facilitation of regeneration and recovery was distinctly accelerated, especially in the motorial function. The generation of motor neuron has been attributed to the interacted action of transcription factors such as *Olig2*, *Isl1*, and *Irx3*. The efficient trigger of *Isl1-Lhx3* in a temporal approach could induce motor neuron, and miR-218 was discovered as a downstream effector (Thiebes et al., 2015).

In addition, myelination of SCs is pivotal in neural repair, and our results showed a greater presence of SCs nuclei in PHBV-miR-218-ASCs-Exo and PHBV-Hypoc-miR-218-ASCs-Exo groups. Herein, we suggested that ASC-secreted exosomes may deliver myelin-promoting factors along with delivering miR-218. Bucan et al. noted that ASC exosomes can be internalized by SCs and significantly increase the proliferation. They first demonstrated that ASC exosomes are enriched with multiple neurotrophic factors, such as glial cell-derived neurotrophic factor (GDNF), fibroblast growth factor-1 (FGF1), brain-derived neurotrophic factor (BDNF), insulin-like growth factor-1 (IGF1), and nerve growth factor (NGF). The beneficial effects triggered by exosomes are closely related to the shuttling of these neurotrophic factors (Bucan et al., 2019). Thus, bioactive molecules related to nerve repair and exosomes delivering high-level miR-218 provide profound support on the axonal outgrowth, SC myelination, and nerve fiber regeneration. In the Hypoc-miR-218-ASCs-Exo group, higher encouraging regeneration was attributed to the lack of sufficient CO_2_ supply, resulting in a modest change of pH-stimulated cell secretion. ASCs were forced to undergo adaptive alterations and enhanced exosome inclusion and loading capacity, and in this context, the concentrated miR-218 and exosomes finally promoted neural repair *in vivo*. Taken together, this study provides new insights into the hypocapnic environment stimulating the secretion of exosomes. Nevertheless, potential manipulatory mechanisms of specific miR-218 from Hypoc-miR-218-ASCs exosomes on the regeneration of neuronal cells or SCs and combinatorial effectiveness of exosomes with other unclarified activators need further exploration.

## Conclusion

The hypocapnia environment could stimulate ASCs to secrete exosomes of smaller size. These exosomes more effectively concentrated the intracellular miR-218 and delivered to recipient cells. As exogenously elements, the special exosomes played as regulators in promoting migratory activity rather than proliferation. *In vivo*, Hypoc-miR-218-ASCs-Exo further accelerated the healing of sciatic nerve injury and promoted the tissue regeneration and motor functional recovery by enhancing myelination. Satisfactory outcomes suggested that environmental modulation becomes an effective treatment of manipulating exosome secretion and function, and the combination of scaffold and exosomes can be novel strategies in peripheral nerve regeneration.

## Data Availability

The original contributions presented in the study are included in the article/Supplementary Material; further inquiries can be directed to the corresponding author.
