# Supplementary material for: Hypocapnia Stimuli-Responsive Engineered Exosomes Delivering miR-218 Facilitate Sciatic Nerve Regeneration
Source: Front Bioeng Biotechnol. 2022 Feb 8;10:825146. doi: 10.3389/fbioe.2022.825146 (PMC8861458; doi:10.3389/fbioe.2022.825146)
Supplement: Supplementary file 1 [file DataSheet1.DOCX]

Supplementary Material

# Supplementary Figures and Tables

## Supplementary Table

# List of Primers Used for qRT-PCR

| Genes | Forward Primers (5’-3’) | Reverse Primers (3’-5’) |
| --- | --- | --- |
| *Gapdh* | CGATCCCGCTAACATCAAAT | GGATGCAGGGATGATGTTCT |
| *Robo1* | GGAGGAAAGATGACGGAGAGC | AGATGTTGGGGTTGCTCCTGA |
| *Robo2* | AAGAAAGAGTTAAGGTGGGTGG | TCTGAAGGACCATCAGGTCC |
| *Sfrp2* | ATCCTGGAGACAAAGAGCAAGACC | TGACCAGATACGGAGCGTTGATG |
| *Dkk2* | GCCAAACTCAACTCCATCAAGTCC | TCTTACTGCCGCCGAAAGCC |
| *miR-218* | ACACTCCAGCTGGGTTGTGCTTGATC | TGGTGTCGTGGAGTCG |
| *U6* | GCTTCGGCAGCACATATACTAAAAT | CGCTTCACGAATTTGCGTGTCAT |

## supplementary Figures


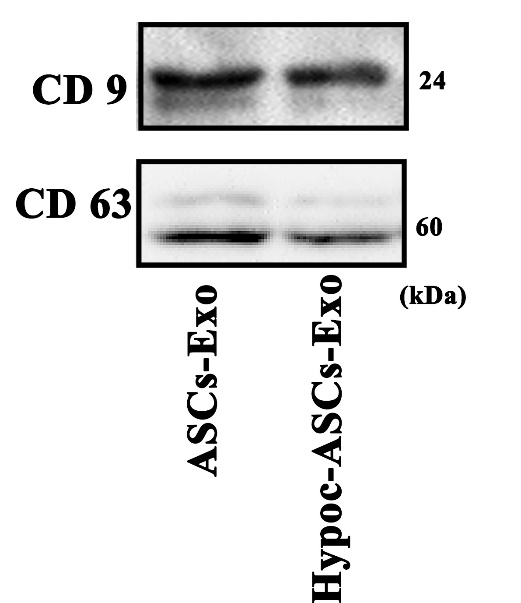


**Supplementary Figure 1.** Specific markers of exosomes (CD 63 and CD9) were confirmed by Western blotting.


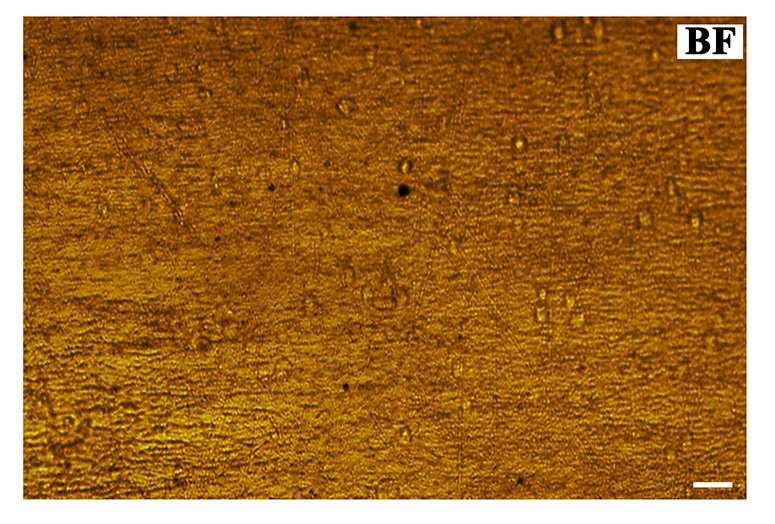


**Supplementary Figure 2.** PC12 cells on the surface of film were shown in bright field (Scale bars= 50 μm).


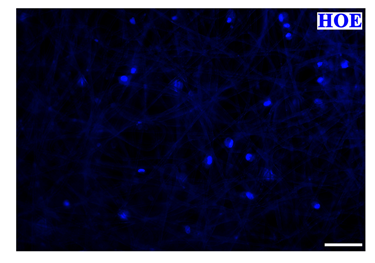


**Supplementary Figure3.** Nuclei of PC12 cells on the surface of film were stained by Hoechst 33342 (blue) (Scale bars= 50 μm)..
